# The emergence and maintenance of sickle cell hotspots in the Mediterranean

**DOI:** 10.1016/j.meegid.2012.06.001

**Published:** 2012-10

**Authors:** Bridget S. Penman, Sunetra Gupta, Caroline O. Buckee

**Affiliations:** a Department of Zoology, University of Oxford, Oxford OX1 3PS, UK; b Center for Communicable Disease Dynamics, Department of Epidemiology, Harvard School of Public Health, Boston, MA 02115, USA

**Keywords:** Sickle cell, Thalassaemia, Malaria, Mediterranean, Epistasis, Metapopulation

## Abstract

Genetic disorders of haemoglobin (haemoglobinopathies), including the thalassaemias and sickle cell anaemia, abound in historically malarious regions, due to the protection they provide against death from severe malaria. Despite the overall spatial correlation between malaria and these disorders, inter-population differences exist in the precise combinations of haemoglobinopathies observed. Greece and Italy present a particularly interesting case study: their high frequencies of beta thalassaemia speak to a history of intense malaria selection, yet they possess very little of the strongly malaria protective mutation responsible for sickle cell anaemia, despite historical migrational links with Africa where high frequencies of sickle cell occur. Twentieth century surveys of beta thalassaemia and sickle cell in Greece, Sicily and Sardinia have revealed striking sickle cell ‘hotspots’ – places where the frequency of sickle cell approaches that seen in Africa while neighbouring populations remain relatively sickle cell free. It remains unclear how these hotspots have been maintained over time without sickle cell spreading throughout the region. Here we use a metapopulation model to show that (i) epistasis between the alpha and beta forms of thalassaemia can restrict the spread of sickle cell through a network of linked subpopulations and (ii) the emergence of sickle cell hotspots requires relatively low levels of gene flow, but the aforementioned epistasis increases the chances of hotspots forming.

## Highlights

► We investigate how sickle cell spreads in a metapopulation containing thalassaemia. ► Epistatically interacting thalassaemias can contain the spread of sickle cell. ► Sickle cell hotspots are promoted by epistasis and low mixing between subpopulations. ► Sickle cell could have challenged Greek and Italian populations for over 2000 years.

## 1 Introduction

The malaria parasite has imposed arguably the strongest evolutionary pressure of any pathogen on human populations. In 1949, JBS Haldane suggested that this selection pressure may have resulted in a high frequency of genetic blood disorders in malaria-endemic populations (Haldane, 1949). This ‘malaria hypothesis’ is now widely accepted to apply to a variety of mutations affecting the alpha or beta globin genes that encode the subunits of haemoglobin (haemoglobinopathies), including those responsible for sickle cell anaemia (Allison, 1954; Williams et al., 2005b); haemoglobin C (Modiano et al., 2001); haemoglobin E (Chotivanich et al., 2002) and the alpha and beta thalassaemias (Hill et al., 1988; Flint et al., 1986). A recent review of the genetics of malaria resistance is provided by Hedrick (2011); Taylor et al. (2012) have also carried out a meta-analysis of the evidence for malaria protection provided by each globin mutation.

Almost every old-world malarious region hosts a range of haemoglobinopathies, but – as reviewed in Flint et al. (1998) – the suite of haemoglobinopathies observed varies between populations. Extensive molecular and epidemiological studies, motivated by the enormous public health significance of these disorders, are starting to elucidate how mutations in the alpha and beta globin genes may interact with one another to affect clinical phenotypes (Kan and Nathan, 1970; Wainscoat et al., 1983a,b; Galanello et al., 1989; Williams et al., 2005a). The haemoglobinopathies thus represent a unique opportunity to compare the relative importance of natural selection, migration and gene-gene interactions (epistasis) in generating spatial genetic variation in human populations.

It has been suggested that heterogeneity in the geographical distribution of haemoglobinopathies results from the relatively recent emergence of strong malaria selection at some point within the last 8000 years, following the increase in human population density associated with the advent of agriculture (Flint et al., 1998). Under this scenario, there simply has not been enough time for population mixing to produce a uniform pattern of mutations. However, genetic spatial heterogeneity can also arise and be maintained in populations through a variety of other evolutionary and ecological processes, (Buckee et al., 2004; Hedrick, 2006; Buckee et al., 2007; Lawton-Rauh, 2008; Cushman et al., 2011; Neto et al., 2011; Lappalainen et al., 2011). We have previously shown that epistatic interactions among the haemoglobinopathies may account for large-scale geographic differences in the relative frequencies of haemoglobinopathies in African and Mediterranean populations (Penman et al., 2009). In Africa, high frequencies of sickle cell (*β*^S^) co-exist with frequencies of alpha thalassaemia that do not exceed 50%, whereas in the Mediterranean, diverse alpha and beta thalassaemic mutations are present, and *β*^S^ is largely absent. We proposed that these contrasting suites of alleles could be maintained by two well-documented genetic interactions: (i) positive epistasis, where coinheriting alpha and beta thalassaemia leads to a less severe blood disorder than that caused by beta thalassaemia alone (Kan and Nathan, 1970; Wainscoat et al., 1983a,b; Galanello et al., 1989; Weatherall and Clegg, 2001a) and (ii) negative epistasis, where coinheriting alpha thalassaemia and sickle cell trait leads to a loss of malaria protection (Williams et al., 2005a; May et al., 2007). The former can allow relatively low frequencies of the thalassaemias to exclude *β*^S^ from a population (Penman et al., 2009); the latter limits the frequency of alpha thalassaemia when *β*^S^ is present (Williams et al., 2005a), or could even allow very high levels of alpha thalassaemia to exclude *β*^S^ (Penman et al., 2011).

**Fig. 1:**
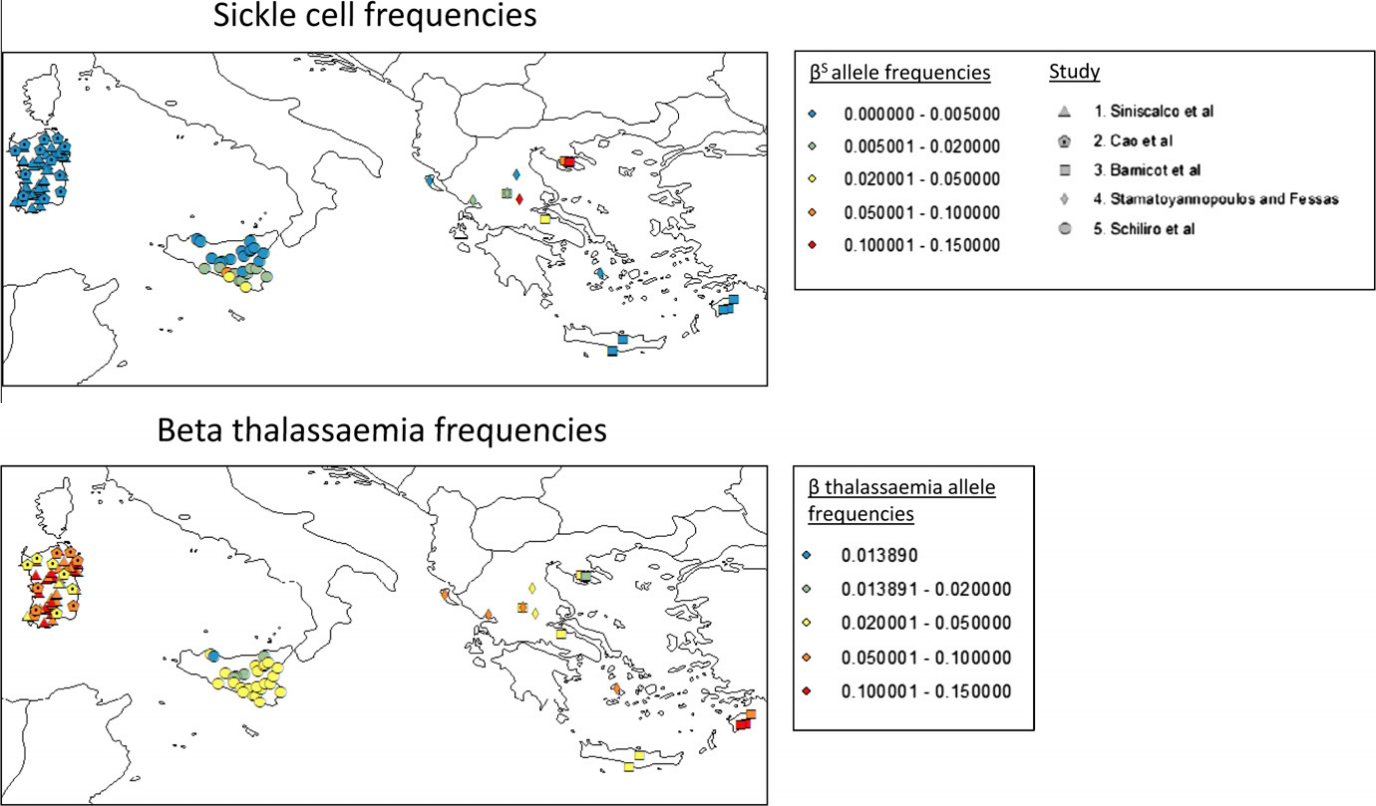
Visualizing five studies of sickle cell and beta thalassaemia in the Mediterranean. This map summarises data from five studies: (Barnicot et al., 1963; Stamatoyannopoulos and Fessas, 1964; Siniscalco et al., 1966; Cao et al., 2008; Schiliro et al., 1986). Each study recorded the number of beta thalassaemia or sickle cell heterozygotes; we have converted these into allele frequencies, but since homozygotes were excluded from the studies shown these allele frequencies may be a slight underestimate. Supplementary Table S1 provides more information about these data. This map was produced using arcGIS10 (For interpretation to colours in this figure, the reader is refered to the web version of this article.).

Although this framework provides a parsimonious explanation for the broad qualitative patterns observed across the two continents, striking local heterogeneities occur in the distribution of blood disorders within populations, which cannot be accounted for within a deterministic framework. Fig. 1 collates the results of five geographical surveys that cover Greece, Sicily and Sardinia (Barnicot et al., 1963; Stamatoyannopoulos and Fessas, 1964; Siniscalco et al., 1966; Schiliro et al., 1986; Cao et al., 2008), and reveals several interesting features: beta thalassaemia is ubiquitous and *β*^S^ is limited in its spatial distribution (as previously observed), yet in a number of ‘hotspots’ the *β*^S^ frequency observed in Greek populations is extremely high (>0.1) and comparable to that seen in Africa. It is unclear how these hotspots have been maintained, and why *β*^S^ has not spread further across the Mediterranean given (i) the history of strong malaria selection in Greece and southern Italy; (ii) the extremely high degree of malaria protection *β*^S^ provides, and (iii) the close migrational ties between Africa, Greece, and Italy. Here, we explore these local patterns of genetic variation in the Mediterranean, and develop a metapopulation model to investigate how processes such as migration and selection impact the local heterogeneity of blood disorders. This follows the successful use of linear metapopulation (‘stepping stone’) population genetic models by Livingstone (Livingstone 1969a, 1976, 1989) to explore the distribution of malaria protective beta globin variants, both in West Africa and across the old world. We confirm that epistatic interactions between the thalassaemias can help keep *β*^S^ frequencies low throughout most of a population. We also show that hotspots of sickle such as those observed in current Mediterranean populations require low levels of gene flow between the hotspot region and the rest of the population if they are to be maintained, but that epistasis increases the range of gene flows at which hotspots are possible. It is widely believed that *β*^S^ came to the Mediterranean from Africa. Our results suggest that *β*^S^ could have first been introduced to the Mediterranean >2000 years before the present.

## 2 Methods

We limit our attention to the three most common haemoglobinopathies in the Mediterranean region (Weatherall and Clegg, 2001b): alpha thalassaemia caused by a single deletion of an alpha globin gene (represented as *α*^+^); beta thalassaemia caused by a mutation which significantly reduces but does not completely eliminate beta globin production (*β*^+^), and the point mutation responsible for sickle cell anaemia (*β*^S^). This scheme captures the most important features of Mediterranean haemoglobinopathies, but necessarily simplifies the situation at the beta globin locus by representing multiple beta thalassaemic mutations with a single variant (see Supplementary material). We consider a metapopulation consisting of 40 subpopulations (demes) linked via migration.

Migration between demes was simulated every generation by letting a certain proportion of each deme (*m*) be assigned allele frequencies equal to the average allele frequencies across all the demes connected to it. Connections between demes followed a predefined network structure (see Supplementary Methods and Watts and Strogatz, 1998). *m* represents ‘gene flow’ in the sense of the proportion of alleles in a population which originated outside of that population, and the chosen value of *m* determines whether the demes represent a small villages or settlements, with strong ties to other villages in the same region, or larger collections of settlements in different regions, with weaker migrational ties.

The frequency of each gene of interest within each deme was calculated using the following standard population genetic equation (see Mandel, 1958) relating the frequency of allele *i* (*p_i_*) in generation *t* + 1 to the frequency of the allele *i* in generation *t*:

$$p_{i(t+1)}=p_{i(t)}.\frac{\sum_{j=1\text{;}k=1\text{;}l=1}^{j=2\text{;}k=3\text{;}l=3}(a_{\mathrm{ijkl}}p_{j}p_{k}p_{l})}{\sum_{i=1\text{;}j=1\text{;}k=1\text{;}l=1}^{i=2\text{;}j=2\text{;}k=3\text{;}l=3}a_{\mathrm{ijkl}}p_{i}p_{j}p_{k}p_{l}}$$

where *i* and *j* represent the frequencies of alleles at the α-globin locus and take values 1 or 2, and *k* and *l* represent the frequencies of allele at the β-globin locus and take values 1,2, or 3. *a_ijkl_* represents the fitness of genotype ‘*ijkl*’. We have chosen to ignore the effects of genetic drift, since all the alleles we are concerned with are under strong selection from malaria. α-globin and β-globin are considered completely unlinked, since the alpha and beta globin clusters occur on different chromosomes in humans.

In order to investigate the effects of epistasis, each genotype was allocated two mortality rates: one associated with the severity of its blood disorder, and one associated with the level of malaria protection it experiences (see Table 1). We converted these mortality rates into a measure of relative fitness (see Supplementary material), and considered two different scenarios: one where positive epistasis between alpha and beta thalassaemia was included, and one where it was not. Negative epistasis between alpha thalassaemia and sickle cell trait was always included, since we have strong evidence that it occurs (Williams et al., 2005a; May et al., 2007), and we wanted to assess the impact of positive epistasis explicitly. Whenever we refer to ‘epistasis’ in the results and discussion, we are referring to positive epistasis between alpha and beta thalassaemia.

At the start of each simulation, all demes were assumed to contain a set starting frequency (*T*) of *α*^+^, and the same starting frequency of *β*^+^. We ran two different versions of the model – in the first, we assumed that *β*^S^ was first introduced 100 generations ago (assuming a generation time of 20 years, this is approximately 2000 years ago), at a frequency of 0.001, into a population containing a fixed proportion of the thalassaemias. For the second scenario, we considered 200 generations (4000 years) of malaria selection, and investigated what happened when *β*^S^ was first introduced at various time points. After the first introduction of *β*^S^ in any simulation, we allowed it to arrive at a frequency of 0.001 in a randomly chosen subset of subsequent generations. The first introduction of *β*^S^ was always into deme 20, and subsequent introductions were always into either 20 or 40, which represent trading posts, or other entry points into the region.

Reported beta thalassaemia frequencies from Mediterranean communities range between 0.014 and 0.19 (Fig. 1). Alpha thalassaemia frequencies are less well-known (see further discussion in the Supplementary material). The studies which made up Fig. 1 have been conducted at different scales: villages, sets of villages, or screening centres serving whole regions – so we do not claim to have a clear idea of the true frequency at a particular spatial scale, and nor do we seek to exactly recapitulate the history of the Mediterranean in our simulations. Nevertheless, we have applied some constraints in an attempt to keep our simulations realistic: we aimed to keep ‘modern day’ beta thalassaemic frequencies below 0.19 and ‘modern day’ alpha thalassaemia frequencies below 0.3 (see Supplementary material). This limited the ranges of starting thalassaemia frequencies we tested.

For the sake of simplicity, we have only included one element of stochasticity in this model: the randomly chosen generations in which sickle cell re-challenges the population. Supplementary Fig. S7 illustrates how results vary over 30 repeats, with different sets of parameters. We considered that this minimum of 30 repeats gave a reliable sense of the emerging patterns. 100 repeats were used to generate each cell of a heatmap.

## 3 Results

### 3.1 Epistasis can restrict the spread of *β*^S^ , despite extensive gene flow

**Fig. 2:**
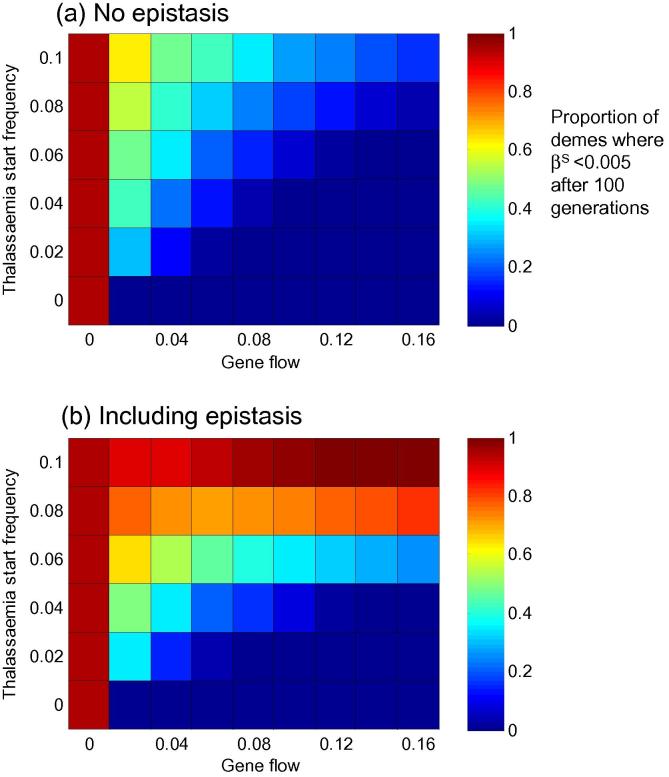
The effect of gene flow and thalassaemia start frequencies on the spread of sickle cell, with and without epistasis. Panels (a) and (b) illustrate the results of a scenario where *β*^S^ was first introduced 100 generations ago, into a population containing fixed (and identical) frequencies of both *α* and *β* thalassaemia (*y* axis). Malaria selection is applied to every deme at a level of 0.005 years^−1^, and after its first introduction, *β*^S^ is assumed to re-challenge the population in 30% of subsequent generations, chosen at random. The colour of each cell in the heatmap indicates the mean proportion of demes where the frequency of *β*^S^ is <0.005 after 100 generations. 100 repeated simulations were used to generate each cell. Supplementary Fig. S5 offers a detailed illustration of the data underlying this figure for thalassaemia starting frequencies of 0.04 and 0.08. (For interpretation of the references to colour in this figure legend, the reader is referred to the web version of this article.)

Figs. 2a and b illustrate how gene flow (*x* axis) and the frequency of thalassaemia present when *β*^S^ arrives (*y* axis) affect the spread of *β*^S^ through the population. So long as the thalassaemias are above a certain threshold when *β*^S^ arrives (0.08 in this case), the inclusion of positive epistasis between alpha and beta thalassaemia can limit its distribution over the next 100 generations. This result echoes our 2009 paper (Penman et al., 2009), but extends our general observation – that a threshold level of epistatically interacting thalassaemias can restrict *β*^S^ – to a network of linked subpopulations, some of which are repeatedly challenged by *β*^S^.

In our 2009 paper, complete competitive exclusion of *β*^S^ by beta thalassaemia was possible, and the ‘winning’ allele was determined by: (i) the relative fitness of beta thalassaemia (which is inherently bound up with its blood disorder severity, malaria protectiveness, and whether or not epistasis with alpha thalassaemia is present) and (ii) the frequency of beta thalassaemia when sickle cell arrives (see Supplementary Fig. S4 and Penman et al., 2009). Within the metapopulation presented here, complete exclusion of *β*^S^ is not possible during the 100 generations we have simulated (the equilibrium point described in Penman et al., 2009 is not reached). However, the average frequency of *β*^S^ in the population after 100 generations is lower in the presence of epistasis than in its absence, and epistasis renders the entire population less susceptible to the effects of increasing the rate of gene flow (Fig. 2 and Supplementary Fig. S5).

Contrary to our expectations, the precise network structure underlying patterns of migration between demes was not a primary determinant of the spread of sickle cell (Supplementary Fig. S7). Whilst in certain circumstances an effect of network structure is discernible (discussed in the supplementary material), its impact was minor in comparison to the starting frequency of thalassaemia, or the level of gene flow between the demes.

How does a 100 generation simulation play out with the starting thalassaemia allele frequencies of Fig. 2? As shown in Supplementary Fig. S6, alpha thalassaemia frequencies increase over the 100 generations, reaching a ‘present day’ frequency of ∼0.28. Beta thalassaemia frequencies on the other hand, actually decline from their starting frequency of 0.08 (although as can be seen in Supplementary Fig. S4, under epistasis they would eventually recover). We can conclude that (i) a thalassaemia frequency of 0.08 100 generations ago need not be associated with unrealistic beta thalassaemia frequencies today, but also that (ii) for *β*^S^ to be kept out of a population, some earlier process (such as long term malaria selection, or simply the rapid spread of thalassaemic alleles in small populations) must have led to high thalassaemia levels before *β*^S^ arrived.

### 3.2 The likelihood of “sickle hotspots” is increased by epistasis but only under low levels of gene flow

As discussed in the introduction, the distribution of *β*^S^ in the Mediterranean is highly discontinuous. Based on the observations in Fig. 1, where the Chalkidhiki peninsular in Greece represents a ‘spike’ of *β*^S^ surrounded by an environment of lower frequencies, we defined a ‘*β*^S^ hotspot’ as a deme with a *β*^S^ frequency >0.06, where the median frequency of *β*^S^ in demes with which it has a direct connection via migration is <0.02. We chose this definition so as to capture the idea of *β*^S^ being contained within a particular region.

**Fig. 3:**
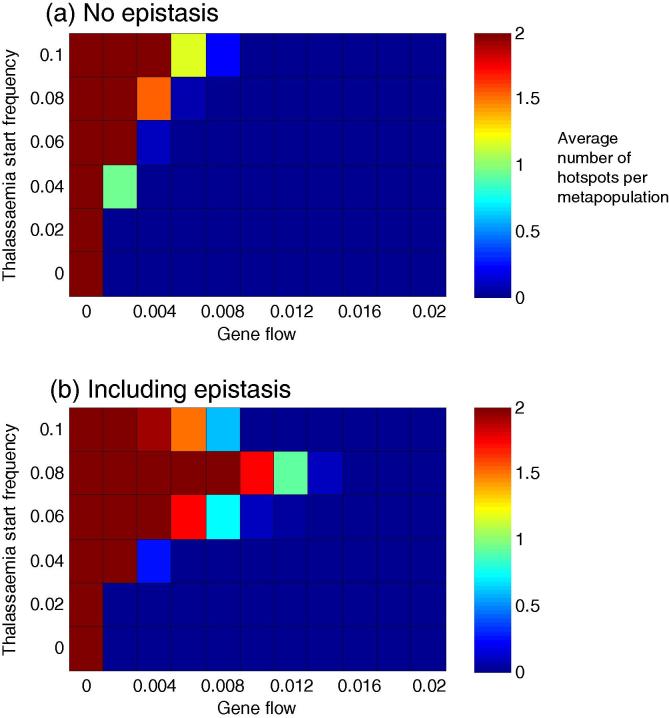
The effect of gene flow and thalassaemia start frequencies on the formation of hotspots, with and without epistasis. Panels (a) and (b) illustrate the results of a scenario where *β*^S^ was first introduced 100 generations ago, into a population containing fixed (and identical) frequencies of both *α* and *β* thalassaemia (*y* axis). Malaria selection is applied to every deme at a level of 0.005 years^−1^, and after its first introduction, *β*^S^ is assumed to re-challenge the population in 100% of subsequent generations. The colour of each cell in the heatmap indicates the average number of hotspots observed per metapopulation over 100 simulations at that parameter combination. (For interpretation of the references to colour in this figure legend, the reader is referred to the web version of this article.)

Fig. 3 illustrates the likelihood of obtaining such hotspots with and without epistasis, for different thalassaemia starting frequencies; levels of gene flow and levels of malaria selection. Two results are apparent: (i) hotspot demes are only possible at relatively low levels of interdeme gene flow, but (ii) the inclusion of epistasis and a threshold starting frequency of thalassaemia increases the parameter space where hotspots are possible. Supplementary Figs. S7 and S8 provide a fuller exploration of the effects of network structure and sickle cell challenge on the formation of hotspots. Once again, network structure appears to be one of the least important factors.

The likelihood of hotspots in the scenario captured in Fig. 3 is maximised when epistasis is present and the starting frequency of alpha and beta thalassaemia is 0.08 (Fig. 3b). This result can be understood when viewed in conjunction with Fig. 2b: at lower thalassaemia starting frequencies, *β*^S^ will occupy a large proportion of the entire network, but at higher thalassaemia starting frequencies, the thalassaemias are so effective at keeping *β*^S^ out that not even a small hotspot can form.

To explore the formation of hotspots further, we developed the pared-down network presented in Supplementary Fig. S9. Two communities with high internal inter-deme gene flow are linked by a connection of much lower gene flow. *β*^S^ is introduced at a high frequency into one deme in community one (simulating the arrival, perhaps, of a population transplanted from Africa). By considering the frequencies of *β*^S^ in the two communities, Supplementary Fig. S9 lets us move away from our somewhat arbitrary definition of a hotspot above, and see that for any chosen ratio of *β*^S^ frequencies in the two communities (e.g. a ratio of 10:1), the inclusion of epistasis allows that ratio to occur at a higher level of gene flow between the communities.

### 3.3 Epistasis allows for a longer history of *β*^S^ challenge in the Mediterranean

**Fig. 4:**
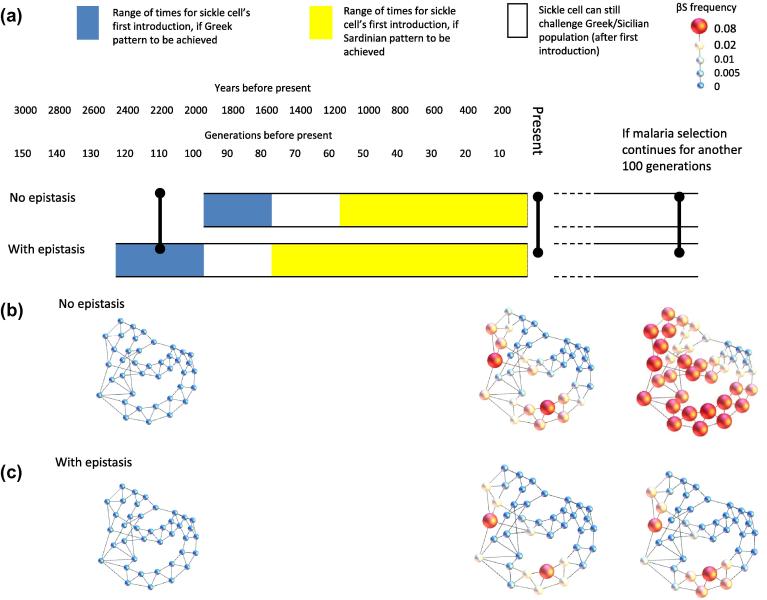
A timeline of sickle cell introduction. In this simulation, the mixing level was set at 0.01, and *β*^S^ was able to challenge the population in 75% of generations after its point of initial introduction. The timeline is based on a generation time of 20 years. The frequencies of both alpha and beta thalassaemia at the beginning of the simulation (200 generations before the present) were 0.02. Thirty repeats were carried out. The Greek and Sardinian patterns were defined as follows: for the Sardinian pattern, the mean proportion of demes with a *β*^S^ frequency <0.005 must be >0.9; for the Greek pattern, the mean proportion of demes with a *β*^S^ frequency <0.005 must be >0.5, and the mean number of hotspots for that entry time must be >0.1. The network pictures illustrate snapshots in one possible time line, when sickle cell was first introduced 120 generations ago with (c) or without (b) epistasis. The colour of each node indicates the frequency of *β*^S^, and the size of each node indicates the intensity of malaria selection experienced by that deme. The network diagrams in this figure were produced using Gephi (Bastian et al., 2009). (For interpretation of the references to colour in this figure legend, the reader is referred to the web version of this article.)

We next explored the temporal scales underlying sickle cell hotspot formation and maintenance in the region. As described in the Section 2, we considered a 200 generation long scenario, in which we tested the outcomes when *β*^S^ was first introduced at different time points. Fig. 4a matches the time of entry of *β*^S^ to approximate present day Sardinian and Greek/Sicilian patterns, for one such timeline. Details of the criteria for each present day pattern are provided in the figure legend. The inclusion of epistasis increases the range of times for the first introduction of *β*^S^ that are consistent with a present day Sardinian scenario. The Greek/Sicilian pattern is associated with a relatively narrow window for the first introduction of *β*^S^, but the presence of epistasis allows this window to occur earlier – implying a longer history of *β*^S^ challenge.

The small window of potential first entry times for *β*^S^ associated with the Greek/Sicilian pattern reflects the transient nature of the hotspots themselves: enough time must pass so that *β*^S^ attains hotspot frequencies, but not so much time that *β*^S^ can spread too much into the surrounding demes.

Figs. 4b and c offer snapshots of different stages of the simulation, for a scenario where the first entry of sickle cell was 110 generations ago. In the presence of epistasis, present day high frequencies of *β*^S^ are limited to its points of entry and their immediate contacts; without epistasis, by contrast *β*^S^ has spread further into the secondary contacts of the original points of entry. If we allowed these populations to continue to evolve over another 100 generations of malaria selection, we arrive at the scenarios shown at the right hand side. Without epistasis, *β*^S^ dominates the population, and will eventually take over completely: genetic heterogeneity is thus a short-lived phenomenon. In the presence of epistasis, however, hotspots can be maintained indefinitely. Gene-gene interactions therefore provide a mechanism for generating genetic heterogeneity in populations that is stable in time and space.

## 4 Discussion

Our model illustrates that a threshold level of epistatically interacting thalassaemias can (i) keep *β*^S^ frequencies very low in most demes in a population, and (ii) extend the circumstances under which *β*^S^ hotspots are possible, within the overall constraint that hotspots can only emerge in relatively self-contained communities with low levels of gene flow to the rest of the population. We propose that a combination of these two effects could have allowed *β*^S^ to challenge the Mediterranean for over two millennia, yet still be restricted to a few isolated regions today. These results confirm our previous large-scale model (Penman et al., 2009), and provide a spatially explicit framework incorporating migration, local heterogeneity and plausible time frames.

Published data on the distribution of haemoglobinopathies in the Mediterranean can be resolved into two main patterns: Greece and Sicily, where *β*^S^ is present and attains pockets of high frequency, and Sardinia, where *β*^S^ is almost completely absent. Explaining the Sardinian pattern is straightforward: Fig. 2 illustrates that, in the presence of positive epistasis between alpha and beta thalassaemia, a threshold level of the thalassaemias can prevent sickle cell from taking over a population – regardless of the level of gene flow between demes within that population. Explaining the hotspots of the Greek/Sicilian population is more challenging: hotspot demes only form within a relatively constrained region of parameter space. We saw in Fig. 4 that the window for the first introduction of *β*^S^ associated with the Greek/Sicilian scenario is small both with and without epistasis, but that epistasis causes it to occur earlier. The interaction between alpha and beta thalassaemia slows the rise in frequency of *β*^S^, delaying both the time taken to reach hotspot levels and its rate of spread out of the introduction deme.

We have illustrated the behaviour of the model at various rates of gene flow, but what is a reasonable degree of mixing to assume between different demes within a metapopulation? Adams and Kasakoff (1976) explored the relationship between endogamy and population size. They found that sub populations in most societies could be described in terms of ‘80% endogamous groups’: a tribe in a pastoralist society; a valley in the highlands of New Guinea; a set of small villages in a peasant society. Levels of endogamy higher than 80% were typically associated with whole ethnic groups. When the gene flow level in our model (*m*) is set at 0.1, our demes are consistent with 80% endogamous groups (assuming 20% of marriages bring in a partner from outside the group, contributing 10% of the genetic material to the next generation). We found that gene flows of 1% or lower were necessary to generate hotspot demes. This implies that the type of deme which can exist as a sickle cell hotspot cannot be a single 80% endogamous local population: instead it may be a relatively closed collection of such 80% groups, with geographical or social barriers that limit genetic exchange with other populations.

Metapopulation approaches are widely used in ecology to model the dynamics of populations in fragmented or spatially heterogeneous habitats (reviewed in Hanski and Gilpin, 1991), and were first applied to the haemoglobinopathies by Livingstone in the form of a ‘stepping stone’ population genetic model. In such a model, demes are ranged along a line, most migration occurs between immediate or secondary neighbours, but there is also the possibility of random long-distance migration (Livingstone 1969a, 1976, 1989). Livingstone considered the generation and maintenance of clines in the frequencies of *β*^S^ in West Africa and beta thalassaemia in Sardinia (Livingstone, 1969a); the overlapping clines of *β*^S^, beta thalassaemia and haemoglobin C in West Africa (Livingstone, 1976), and the interaction between *β*^S^, beta thalassaemia, haemoglobin C and haemoglobin E on an inter-continental scale (Livingstone, 1989). This series of papers demonstrated that the global distribution of the malaria protective haemoglobinopathies is a product of human migration and gene flow as well as malaria selection. In response to Livingstone’s work, Fix (see review: Fix, 2003 and chapter 4 of Fix, 1999) considered other factors which could lead to the rapid accumulation and spread of malaria protective haemoglobin variants, including kin-structured migration (where families were assumed to migrate together, concentrating the import of particular alleles) and a group selection model in which demes with insufficient frequencies of malaria protective alleles risked becoming extinct.

Both Fix’s and Livingstone’s work aimed to account for present day allele frequencies and clines within realistic historical timescales. Enhancing the spread of malaria protective variants (by assuming long distance gene flow or the other processes mentioned above) was necessary in order that present day patterns could be achieved within the hundreds of generations that malaria is thought to have exacted significant mortality on human populations. The work we have presented here is concerned with the opposite problem: how could a high frequency of *β*^S^ have accumulated in a population under malaria selection, yet not spread over the course of ∼100 generations? Our model differs from those of Livingstone and Fix in that we considered alleles at both the alpha and beta globin loci, not beta globin alone, and we sought to understand the role of epistasis in creating present day patterns. Epistasis affects the rate of spread of sickle cell, but it can only help to generate hotspots if gene flow in and out of the hotspot population is already low.

Unlike Livingstone’s models, Fix developed models considering whole numbers of individuals. This modification made it easier to consider both kin structured migration and genetic drift. Livingstone also used a model of a finite yet growing population to demonstrate that a founder effect coupled with rapid population expansion could retain the *β*^S^ gene even in the absence of malaria selection (Livingstone, 1969b). In future efforts to understand the micro-heterogeneity of the haemoglobinopathies in the Mediterranean, spatially explicit individual based models with greater stochasticity and more demographic detail than the framework presented here are likely to prove extremely informative.

Fig. 4c shows that the presence of epistasis can contain the spread of sickle cell indefinitely. However, the population in the third panel of 4c has alpha thalassaemia frequencies of ∼0.57. Since such frequencies are higher than those reported in the twentieth century Mediterranean, we did not consider it a realistic present day outcome. The relatively low frequencies of alpha thalassaemia reported in the Mediterranean are discussed further in the supplementary material; they are a significant constraint on the outcomes possible within the model, and it is unfortunate that we do not have estimates of alpha thalassaemia frequencies at a comparable geographical scale to those for beta thalassaemia. Three of the studies in Fig. 1 (Barnicot et al., 1963; Stamatoyannopoulos and Fessas, 1964; Siniscalco et al., 1966) commented on the patterns of *β*^S^ and beta thalassaemia in the Mediterranean, and suggested that these two beta globin variants may act to exclude one another from populations. Our model is consistent with the conclusion that *β*^S^ in the Mediterranean has been restricted by evolutionary interactions between genes – however, we have shown that a combination of both alpha and beta thalassaemia can be more effective at excluding *β*^S^ than beta thalassaemia alone. Whether or not a beta thalassaemic allele can out-compete *β*^S^ on its own depends on how much malaria protection it offers and how severe a blood disorder it causes. In Supplementary Fig. S4 we illustrate the range of hypothetical beta thalassaemic alleles that can outcompete *β*^S^, and show how this range is extended by the inclusion of epistasis with alpha thalassaemia. It remains a theoretical possibility that the beta thalassaemia found in the Mediterranean has the necessary properties to outcompete *β*^S^ alone, but we have not considered this possibility here, since we wished to focus on the potential population genetic impact of epistasis.

A recent survey of *β*^S^ and beta thalassaemia within the Chalkidhiki peninsular (Kalleas et al., 2012) provides more detail about the modern day pattern of beta globin variants in one of the Greek *β*^S^ hotspots. It is striking that the distribution of *β*^S^ is highly patchy within the peninsular (see Fig. 1 of Kalleas et al.), whilst thalassaemia is present even in (historically non-malarious) mountainous regions – presumably a signature of gene flow within the peninsular. The most intriguing observation in this study relates to the beta thalassaemic mutations present in the Chalkidhiki peninsular alongside *β*^S^. Throughout most of the Greek mainland, the IVS-I-110 beta thalassaemic mutation is more common than the codon 39 beta thalassaemic mutation, but in Chalkidhiki the pattern is reversed. Kalleas et al. note that there is a second region (in Central Greece) where codon 39 exceeds IVS-I-110, and where *β*^S^ is also present. However, codon 39 also predominates in Sardinia, which is *β*^S^ free. Kalleas et al. point out that the relative geographical isolation of all of these regions may have contributed to their particular genetic patterns. Nevertheless, it is also tempting to speculate that the suite of beta thalassaemic mutations predominating in a particular region may have made particular populations more or less susceptible to sickle cell invasion. For this paper, we represented all beta thalassaemia in the Mediterranean with a single, relatively severe *β*^+^ mutation (see Section 2 and Supplementary material). In reality, codon 39 (which, as we just noted, is very common in Sardinia) is a more severe mutation than IVS-I-110 (see Supplementary material and Weatherall and Clegg, 2001a). Allowing for the diversity of beta thalassaemia in the Mediterranean in future modeling work may shed more light on the *β*^S^ question.

Sickle cell anaemia in Greece is caused by the Benin haplotype (Boussiou et al., 1991), one of several different chromosomal backgrounds on which the *β*^S^ mutation is found. This haplotype is generally believed to have originated in central West Africa, but the timing of its evolution and spread is unclear. Pagnier et al. (1984) speculate that the Benin haplotype spread to North Africa along trans-Saharan trade routes via camel caravans from the third century CE onwards. Depending on when and where *β*^S^ emerged in Africa, however, it could have been imported into Greece and Sicily via Carthaginian slave traders from 650 BCE; Ancient Greek and Roman settlements in North Africa, or the later Arab conquest of Sicily by the ninth Century CE (dates from the Penguin Atlas of World History by Kinder and Hilgemann – see especially pages 39 and 119). The earliest evidence of *β*^S^ from a population known to be in contact with the Ancient Mediterranean comes from a skeleton containing fossilized sickle erythrocytes, excavated from an island in the Persian Gulf that was colonised by Alexander the Great. This skeleton was dated from 130+/−80 years BCE (Salares et al., 2004).

Are there any specific historical reasons for the contrasting patterns observed in Sardinia, Greece and Sicily? Siniscalco et al. (1966) note the relative isolation of the Sardinian interior: “Ancient Greek colonisation was, in fact, limited to Olbia (the northern portion of the island), while the Romans and Carthaginians only exploited the coastal regions for grain”. Such isolation is in keeping with the first introduction of *β*^S^ to Sardinia occurring relatively late – although, importantly, we do not require that Sardinia was never exposed to *β*^S^. In the case of Sicily, Schiliro et al. (1986) point out that most *β*^S^ on the island occurs in coastal regions that were the sites of Ancient Greek colonisation, suggesting that *β*^S^ came to Sicily via Greece. The founders of the ancient Sicilian city states of Syracruse and Gela came from Corinth, Rhodes and Crete – none of which are present day Greek sickle cell hotspots – but it is entirely possible that the Greeks precipitated the spread of sickle cell in their Sicilian settlements by bringing in African slaves. As for Greece itself, Stamatoyannopoulos et al. do not posit any specific historical events as the reason for the Greek pattern of *β*^S^, but they do note that *β*^S^ seems only to be found in regions of high malaria selection. However, given that malaria selection must have been high enough in other areas to elevate the frequency of thalassaemia, we consider it unlikely that the *β*^S^ hotspot pattern is due solely to insufficient malaria pressure in non-hotspot regions.

The models presented here highlight the importance of a low level of gene flow for the formation of *β*^S^ hotspots. A pertinent historical question, then, is whether the various *β*^S^ foci have had any particular reason for isolation. In the Greek cases, the geography of Greece itself (valley communities separated by mountain ranges) could be a factor. The Cholomondas mountains in the central part of Chalkidhiki may have limited gene flow from the coastal communities at the tip of the peninsular, where *β*^S^ is at its highest.

Overall, we have demonstrated that epistasis enhances the ability of thalassaemias to keep *β*^S^ from taking over a population. The formation of *β*^S^ hotspots requires generally low levels of gene flow, but epistasis extends the range of gene flow values where hotspots are possible. Given the potential >2000 year window of *β*^S^ challenge in the Mediterranean and the high degree of malaria protection *β*^S^ offers, the circumscription of its present day distribution suggests something has acted to curtail its spread. We posit that epistasis is an important contributor to this unknown force.

## Figures and Tables

**Table 1:** The relative susceptibility to death from malaria and blood disorder mortality rates assigned to each genotype, with and without epistasis. We wished to determine the influence of positive epistasis between alpha and beta thalassaemia on the generation of *β*^S^ hotspots in the Mediterranean region. The unbracketed figures in Table 1 include this epistasis; the bracketed figures do not include this epistasis. A negative epistatic interaction between alpha thalassaemia and sickle cell trait was always present (see the *β*^S^*β* column), based on a combination of observations by Williams et al. (2005a), May et al. (2007). More details about the values chosen in this table are given in the Supplementary Information, Section 1.

	*β β*	*β*^+^*β*	*β*^S^*β*	*β*^+^*β*^+^	*β*^S^*β*^S^	*β*^+^*β*^S^
*Blood disorder*						
*αα*/*αα*	0.04	0.0405	0.04	0.2	Lethal	Lethal
*α*-/*αα*	0.04	0.04	0.04	0.05 (0.2)	Lethal	Lethal
*α*-/*α*-	0.0415	0.04	0.0415	0.042 (0.2)	Lethal	Lethal

*Malaria susceptibility*						
*αα*/*αα*	1	0.5	0.06	0.5	Lethal	Lethal
*α*-/*αα*	0.85	0.5	0.11	0.5	Lethal	Lethal
*α*-/*α*-	0.6	0.5	0.9	0.5	Lethal	Lethal
