# Supplementary material for: The emergence and maintenance of sickle cell hotspots in the Mediterranean
Source: Infect Genet Evol. 2012 Oct;12(7):1543–50. doi: 10.1016/j.meegid.2012.06.001 (PMC3438445; doi:10.1016/j.meegid.2012.06.001)

Supplementary information

1. **Supplementary Methods and Model Assumptions**

Mortality rates

Table 1 illustrates the mortality rates that were assumed for each genotype, with or without (bracketed) positive epistasis between alpha and beta thalassaemia. We always included a negative interaction between alpha thalassaemia and sickle cell trait (see the β^S^β column), based on a combination of observations by Williams *et al* (2005a) and May *et al* (2007). Specifically, the malaria protectiveness of the αα β^S^β, αα^+^ββ and αα^+^β^S^β genotypes were based on estimates from May *et al* (2007), the malaria protectiveness of α^+^α^+^β^S^β is based on Williams *et al* (2005a) and the malaria protectiveness assigned to α^+^α^+^ββ represents a compromise between estimates from the two studies.

The May *et al* estimate for the malaria protectiveness of the αα^+^ββ genotype is relatively low compared to Williams *et al* (2005a). Reported alpha thalassaemia frequencies in the Mediterranean are lower than those from sub Saharan Africa and parts of Asia. We sought to keep alpha thalassaemia from gaining too high a frequency over the 100 generations of our simulation, so opted for the less protective estimate. Later in the supplementary material we consider potential issues surrounding the true frequencies of alpha thalassaemia in the Mediterranean.

We assumed that homozygous α^+^ thalassaemia and heterozygous β^+^ thalassaemia carry a slight blood disorder cost. Whilst generally regarded as benign conditions in modern populations, the slight anaemia associated with these genotypes may well have exacted a cost historically. Hedrick estimates that a slight cost to homozygous α^+^ thalassaemia must keep α^+^ from drifting to higher frequencies in populations that are not under malaria selection (Hedrick, 2011b). As we have discussed previously (Penman, 2011), including a slight cost to α^+^ increases the range of parameter space for which β^S^ and alpha thalassaemia can coexist as they appear to in Africa.

In Table 1 we assumed that all forms of β^+^ thalassaemia offer 50% protection against death from malaria, and that homozygous β^+^ causes a severe disease in the absence of alleviation by alpha thalassaemia. There are no good estimates in the literature of the malaria protectiveness of beta thalassaemia – later in the supplementary material we discuss the likely properties of beta thalassaemia, and illustrate the effect of epistasis on a range of hypothetical beta thalassaemic alleles.

Relative fitnesses and generation times

By combining the mortality rates given in table 1, we can calculate a total mortality rate for each genotype, for a given level of malaria selection. For example, under a regime where malaria was assumed to exact an excess mortality of 0.005 years^-1^ on the population, the wild type total mortality rate would be 0.04+(1x0.005) = 0.045 years^-1^. The inverse of that total mortality rate gives us the average life expectancy for that genotype (1/0.045=22 years). To calculate the relative fitness of a given genotype we took the ratio of the life expectancy of that genotype to the longest life expectancy in the population. This is a simple way to combine our assumptions about the risks of dying from either a blood disorder or malaria (as outlined in Table 1) into a fitness value that can be incorporated in a standard population genetic framework.

In the main text we have assumed a generation time of 20 years, in order to match our results to a historically reasonable timeline. We have also assumed that malaria exerts an excess mortality of 0.005 years^-1^, equivalent to assuming that malaria is responsible for ~11% of total mortality in the population. Since we have not considered demographic processes in detail, our timelines should not be viewed as an attempt to precisely recapitulate Mediterranean history - we are interested in relative rather than absolute introduction times for β^S^, and in demonstrating how different processes affect the plausibility of different timelines.

Migration networks

In order to model migratory connections between demes, we considered each deme as a node on a ring-shaped network. As a baseline, each deme was connected to its 4 nearest neighbours (see figure S2). In order to create different networks, we took the first two of each deme’s connections in turn and rewired them to randomly chosen partners with probability ‘*p*’. This follows a method described in Watts and Strogatz, (1998). As *p* increases, the average shortest path length between any two demes in the network decreases. Unless otherwise indicated in the main text, figures were produced using a network where p=0.1. Later in the supplementary material we explore the effects of varying *p*.

Assumptions about beta thalassaemia and epistasis

The β^+^ included in our model is intended to represent a ‘typical’ Mediterranean beta thalassaemic allele, based on the fact that of the common beta thalassaemic mutations in Greece and Italy, many are relatively severe (IVS-I-110 G🡪A and IVS-II-745 C🡪G are severe β^+^ alleles; CD39 C🡪T , IVS-I-1G🡪A and CD 6 –A are β^0^ alleles - reviewed in Weatherall and Clegg, 2001a), and cause disease when coinherited with each other or with sickle cell trait. In reality, not all beta thalassaemic phenotypes in the Mediterranean will be able to be alleviated by alpha thalassaemia to the same extent - our model is intended simply to demonstrate that such alleviation (i) could have population genetic consequences, and (ii) opens up new possibilities as to the history of sickle cell’s introduction to the region.

As stated in the introduction, positive epistasis between alpha and beta thalassaemia exists such that when the two disorders are co-inherited, the resulting phenotype can be less severe than had beta thalassaemia been inherited alone. The thalassaemias result from a reduction in the rate of production of either the alpha or beta subunit of haemoglobin: α^+^ and β^+^ mutations cause partial loss of globin production from a chromosome, α^0^ and β^0^ cause complete loss. Many of the adverse symptoms of these disorders derive from the accumulation of whichever subunit is still being produced at the normal rate, so inheriting two thalassaemic disorders can balance out chain synthesis, leading to a less severe clinical course. There is clear evidence that thalassaemia major caused by homozygosity or compound heterozygosity for beta thalassaemic mutations can be alleviated by homozygous α^+^ thalassaemia (Galanello *et al*., 1989), and some suggestion it may be able to be alleviated by heterozygous α^+^ thalassaemia (Wainscoat *et al*., 1983a,b). In this study we have assumed that both homozygous and heterozygous α^+^ thalassaemia offer substantial alleviation of the severity of β^+^β^+^ thalassaemia, so as to assess the maximum population genetic impact of this kind of positive epistasis.

We have assumed a constant ‘malaria protective effect’ of beta thalassaemia, enjoyed by all beta thalassaemic genotypes and unaffected by the coinheritance of alpha thalassaemia. As discussed in our 2009 paper, it is necessary for the compound thalassaemic genotypes to retain a degree of malaria protection if alpha and beta thalassaemia are to work together to exclude β^S^ from a population. We still understand very little about the mechanism of malaria protection operating in the thalassaemias, and if it is linked to the severity of the blood disorder suffered by the individual. Here, as in Penman *et al*., (2009) we are assuming that this malaria protection is at least partly independent of blood disorder severity. Studies have shown that the membrane abnormalities of beta thalassaemia heterozygotes appear to be retained in cells with both alpha and beta thalassaemia (Knox-Macaulay *et al*., 1972), despite the compound thalassaemic cells having a better survival rate (due, presumably, to their more balanced globin chain synthesis). Unless the malaria protection afforded by the thalassaemias hinges entirely on globin chain balance, it is entirely plausible that compound thalassaemic individuals should retain some degree of malaria protection.

In supplementary information section 3 we explore the properties of beta thalassaemia, demonstrating that the range of hypothetical beta thalassaemic alleles capable of excluding an invading β^S^ allele is substantially enhanced by the inclusion of positive epistasis.

1. **Studies of β^S^ and the thalassaemias in the Mediterranean**

Table S1 details the studies which we used to produce the map in figure 1. Since these employ a range of different approaches we have not pooled all of the data together, but instead consider the patterns observed within each separate study. We have also indicated the proportion of sites where β^S^ frequencies <0.005 or >0.06 since these arbitrary measures formed the basis of one of the metrics used in the main text.

| Study | Area | Number of sites observed | Mean number of individuals surveyed at each site | Proportion of sites where β^S^<0.005 | Proportion of sites where β^S^>0.06 | Notes |
| --- | --- | --- | --- | --- | --- | --- |
| Barnicot *et al*., 1963 | Whole of Greece | 10 | 75 | 0.5 | 0.2 | These 10 populations were from towns and villages in Crete and Rhodes, and the Greek mainland (Thessaly, Central Greece and Chalkidhiki). The regions studied were chosen because local knowledge suggested a high incidence of thalassaemia or sickle cell. In 8 of the 10 sites studied, only school children were sampled; in 2 both adults and children were sampled. Almost all of the samples were from males*. HbA_2_ levels were assessed in samples thought to be thalassaemic (either due to a positive one tube osmotic fragility test, or the presence of abnormalities in blood films); the presence of elevated HbA_2_ was used to diagnose carriers of beta thalassaemia. Potential sickle cell carriers were identified by exposure of the blood sample to sodium metabisulphite. |
| Stamatoyannopoulos and Fessas (1964) | Whole of Greece | 6 | 430 | 0.67 | 0.16 | Some of the samples come from collections of villages (e.g. the Corfu sample comes from 7 villages in the North of Corfu), others were from towns. Only males were studied*. All were school children apart from the Corfu sample which also included male adults. Some of the samples noted that particular subsets of individuals were likely to have experienced different levels of malaria endemicity in the local area, but in order to create figure 1 we pooled the results at each individual geographical location. The methods used in this study to identify 'thalassaemia trait' will have detected both beta thalassaemia heterozygotes and individuals carrying alpha thalassaemia with a noticeable phenotype (probably homozygotes for a mild alpha thalassaemic deletion) - but the authors only note alpha thalassaemics in their Petromagoula sample. We have eliminated these definite alpha thalassaemics from the estimates used to create figure 1. |
| Siniscalco *et al*., 1966 | Italy : Sardinia | 45 | 127 | Did not test for β^S^, but it was generally accepted that β^S^ is extremely rare in Sardinia (cases of sickle cell anaemia were scarce) | | A random sample of schoolboys from 52 villages were investigated. Beta thalassaemia carriers were identified by a positive one tube osmotic fragility test (OTOFT). This test is not specific for beta thalassaemia,- it can also detect iron deficiency anaemia and alpha thalassaemia. Siniscalco *et al* tested a subset of their OTOFT positive samples, and found that 96% of them had elevated HbA2, so concluded that most of their OTOFT positive results must represent beta thalassaemia carriers. The frequencies presented in figure 1 may, however, be an over estimate as a result of this method. |
| Cao *et al.,* 2008 | Italy: Sardinia | 13 | 4868 | 1 | 0 | This survey was carried out at regional thalassaemia screening centres, possibly smoothing out any patterns that might have been observed at the individual village level. The individuals screened in this study were 13-14 year olds. Beta thalassaemia carriers were identified by raised HbA_2_ levels, and sickle cell carriers by a sickling test. |
| Schiliro *et al*, 1986 | Italy: Sicily | 27 | 361 | 0.55 | 0.04 | This survey was carried out on primary school children from a range of cities and towns. The presence of beta thalassaemia was confirmed by detecting elevated HbA_2_ levels. Sickle cell trait individuals were identified using the metabisulphite sickling test. |

*Some of these studies also surveyed glucose-6-phosphate dehydrogenase deficiency, so focused on males – but there is no reason to suppose that the frequency of β^S^ or beta thalassaemia should differ between males and females.

**3. Realistic thalassaemia frequencies**

Whilst the studies listed in table S1 give us some idea of the sorts of frequencies beta thalassaemia may have reached in the 20th century Mediterranean, studies of alpha thalassaemia at a similar geographical scale are lacking. That there is alpha thalassaemia in this region is not in question, but estimates for Greece come from studies carried out in hospitals in major cities (Kanavakis *et al*., 1986) and therefore do not reflect the frequencies alpha thalassaemia may reach in an individual region. As for Sardinia, Pirastu *et al* (1982) used DNA based methods to diagnose the presence of a singly deleted alpha globin gene, and found that the frequency of this mutation was 0.18 in a random sample of Sardinian blood donors - but again this does not capture local heterogeneity.

Cao's thalassaemia survey of 13 centres across Sardinia (Cao *et al*., 2008) recorded the numbers of individuals displaying microcytosis alongside a low mean cellular haemoglobin level, normal iron levels and normal levels of HbA_2_ : a combination of traits which is characteristic of alpha thalssaemaia. However, this phenotype alone does not tell us whether these indviduals were homozygous or heterozygous for particular alpha thalassaemic mutations. Frequencies of this phenotype observed by Cao *et al* (2008) in Sardinia range from 0.0906 in Tempio to 0.372 in Oristano. For the purposes of this modeling exercise we took a gene frequency of 0.3 as a maximum realistic ‘present day’ Mediterranean frequency of alpha thalassaemia. This is intended to represent a frequency it may have reached in an area of high malaria selection, not the average frequency across the whole region. Compared to the frequencies alpha thalassaemia reaches in malarious areas elsewhere in the world (Williams *et al*., 2005a; Fodde *et al*., 1988), 0.3 is relatively conservative.

The ‘present day’ frequencies of alpha thalassaemia in the figures presented in this paper did not exceed 0.3, except in two instances. In the the very top of the heatmap in figures 2 and 3, if the starting thalassaemia level was 0.1, and the level of malaria selection was 0.005 years^-1^ then after 100 generations, alpha thalassaemia will have reached a frequency of 0.32 in the presence of epistasis. Additionally, in the timeline of figure 4, the maximum frequency of alpha thalassaemia in any individual deme after 200 generations in the presence of epistasis is 0.309.

**4. Supplementary results**

Equilibria

Depending upon the time at which β^S^ is introduced, the mortality scenario outlined in table 1 is capable of producing the two equilibrium outcomes discussed in our earlier paper (Penman *et al* 2009): namely a ‘Sub Saharan African’ outcome where β^S^ coexists with alpha thalassaemia, but the frequency of alpha thalassaemia is kept below 0.5, and a ‘Mediterranean’ outcome where β^S^ is excluded, but the thalassaemias are maintained. Neither of these equilibria are reached during the limited number of generations that we simulated here, but figure S3 illustrates them for the sake of comparison.

The properties of beta thalassaemia

Hypothetically there could exist β^+^ alleles which are sufficiently fit to exclude β^S^ on their own, without any assistance from alpha thalassaemia. Indeed, observing the abundance of beta thalassaemia and lack of β^S^ in Sardinia, other authors have speculated that there could be competitive exclusion between these mutations. (Barnicot *et al*., 1963; Stamatoyannopoulos and Fessas, 1964; Siniscalco *et al*., 1966). Figure S4 explores this, defining beta thalassaemic alleles by their malaria protectiveness and the severity of their blood disorder in the homozygous state. Let us define ‘plausible’ β^+^ alleles as those which are fit enough to exclude β^S^ from a moderate starting frequency (e.g 0.01-0.1), but are not so fit that they can exclude β^S^ from a starting frequency <0.01 (after all, if β^+^ were that fit we would surely expect to see more of it competing with β^S^ in sub-Saharan Africa). Epistasis extends this range of plausible β^+^ alleles considerably, to include more severe and less malaria protective possibilities.

The effect of network structure

We generated 30 networks at each of 8 different levels of p, and tested them over 100 generations, with the same level of malaria selection and probability of sickle cell introduction. Figure S7 illustrates the results, for 2 different levels of gene flow and 2 different starting frequencies of the thalassaemias. The only obvious effects of network structure are in panels (a) and (b), with 10% mixing between demes. If conditions are such that β^S^ takes ≈100 generations to spread through the whole network - as occurs in panel a without epistasis (blue) or panel b with epistasis (red), then there is a negative correlation between the proportion of random connections in the network (p) and the proportion of β^S^ free demes. Unsurprisingly, the better connected the network, the more easily β^S^ can spread. If, however, conditions are such that β^S^ is being kept out of most of the network by epistasis (panel a, with epistasis, red), then there appears to be a slight positive correlation between the proportion of random connections in the network and the proportion of β^S^ free demes. Perhaps, when β^S^ is competing with a high level of epistatically interacting thalassaemias, it has more success spreading amongst local demes that are interconnected in an ordered fashion, rather than dispersing along more random connections. Positive feedbacks between such interconnected demes (at low levels of p) could help boost β^S^ levels despite competition from the thalassaemias.

In addition to the proportion of β^S^ free demes, figure S6 also illustrates the effect of network structure on the generation of hotspots. Hotspots only appear in panel (d) - reflecting the limited range of parameter space in which they occur. Network structure does not appear to have a dramatic influence on the generation of hotspots.

**References**

Barnicot, N.A., Allison, A.C., Blumberg, B.S., Deliyannis, G., Krimbas, C., Ballas, A., 1963. Haemoglobin types in Greek populations. Ann. Hum. Genet. 26, 229-236.

Cao, A., Congiu, R., Sollaino, M.C., Desogus, M.F., Demartis, F.R., Loi, D., Cau, M., Galanello, R., 2008. Thalassaemia and glucose-6-phosphate dehydrogenase screening in 13- to 14-year-old students of the Sardinian population: Preliminary findings. Community Genetics 11, 121-128.

Fodde, R., Losekoot, M., Van, d.B., Oldenburg, M., Rashida, N., Schreuder, A., Wijnen, J.T., Giordano, P.C., Nayudu, N.V.S., Meera Khan, P., Bernini, L.F., 1988. Prevalence and molecular heterogeneity of alpha+ thalassemia in two tribal populations from Andhra Pradesh, India. Hum. Genet. 80, 157-160.

Galanello, R., Dessi, E., Melis, M.A., Addis, M., Sanna, M.A., Rosatelli, C., Argiolu, F., Giagu, N., Turco, M.P., Carace, E., Pirastu, M., Cao, A., 1989. Molecular analysis of β(o)-thalassemia intermedia in Sardinia. Blood 74, 823-827.

Hedrick, P.W., 2011b. Selection and mutation for α thalassemia in nonmalarial and malarial environments. Annals of Human Genetics 75, 468-474

Kanavakis, E., Tzotzos, S., Liapaki, A., 1986. Frequency of α-thalassemia in Greece. American Journal of Hematology 22, 225-232.

Knox-Macaulay, H.H., Weatherall, D.J., Clegg, J.B., Bradley, J., Brown, M.J., 1972. The clinical and biosynthetic characterization of αβ-thalassaemia. Br. J. Haematol. 22, 497-512.

May, J., Evans, J.A., Timmann, C., Ehmen, C., Busch, W., Thye, T., Agbenyega, T., Horstmann, R.D., 2007. Hemoglobin variants and disease manifestations in severe falciparum malaria. JAMA 297, 2220-2226.

Penman, B.S., Pybus, O.G., Weatherall, D.J., Gupta, S., 2009. Epistatic interactions between genetic disorders of hemoglobin can explain why the sickle-cell gene is uncommon in the Mediterranean. PNAS 106, 21242-21246.

Penman, B.S., Habib, S., Kanchan, K., Gupta, S., 2011. Negative epistasis between α+ thalassaemia and sickle cell trait can explain interpopulation variation in South Asia. Evolution 65, 3625-3632

Pirastu M., Lee K.Y., Dozy A.M., Kan Y.W., Stamatoyannopoulos G., Hadjiminas M.G., Zachariades Z., Angius A., Furbetta M., Rosatelli C., Cao A., 1982. Alpha-thalassemia in two Mediterranean populations. Blood 60, 509-12.

Schiliro, G., Di Gregorio, F., Romeo, M.A., 1986. Incidence of hemoglobin S carriers in Sicily. Hemoglobin 10, 95-99.

Siniscalco, M., Bernini, L., Fillipi, G., Latte, B., Meera Kahn, P., Piomelli, S., Rattazzi, M., 1966. Population Genetics of Haemoglobin Variants, Thalassaemia and Glucose-6-Phosphate Dehydrogenase Deficiency, with Particular Reference to the Malaria Hypothesis. Bull. Wld Hlth Org , 379-393.

Stamatoyannopoulos, G., Fessas, P.H., 1964. Thalassaemia, Glucose-6-Phosphate Dehydrogenase Deficiency, Sickling and Malarial Endemicity in Greece: a study of five areas. Brit. Med. J 1, 875-879.

Wainscoat, J.S., Kanavakis, E., Wood, W.G., 1983a. Thalassaemia intermedia in Cyprus: The interaction of α and β thalassaemia. Br. J. Haematol. 53, 411-416.

Wainscoat, J.S., Old, J.M., Weatherall, D.J., Orkin, S.H., 1983b. The molecular basis for the clinical diversity of β-thalassaemia in Cypriots. Lancet 1, 1235-1237.

Watts, D.J., Strogatz, S.H., 1998. Collective dynamics of 'small-world networks. Nature 393, 440-442.

Weatherall, D.J., Clegg, J.B., 2001a. The Thalassaemia Syndromes, 4 ed. Blackwell Science, Oxford, United Kingdom.

Williams, T.N., Mwangi, T.W., Wambua, S., Peto, T.E.A., Weatherall, D.J., Gupta, S., Recker, M., Penman, B.S., Uyoga, S., Macharia, A., Mwacharo, J.K., Snow, R.W., Marsh, K., 2005a. Negative Epistasis between the malaria-protective effects of alpha+ thalassemia and the sickle cell trait. Nat. Genet. 37, 1253-1257.

**Figure S1: A schematic representation of the processes occurring in each deme every generation**


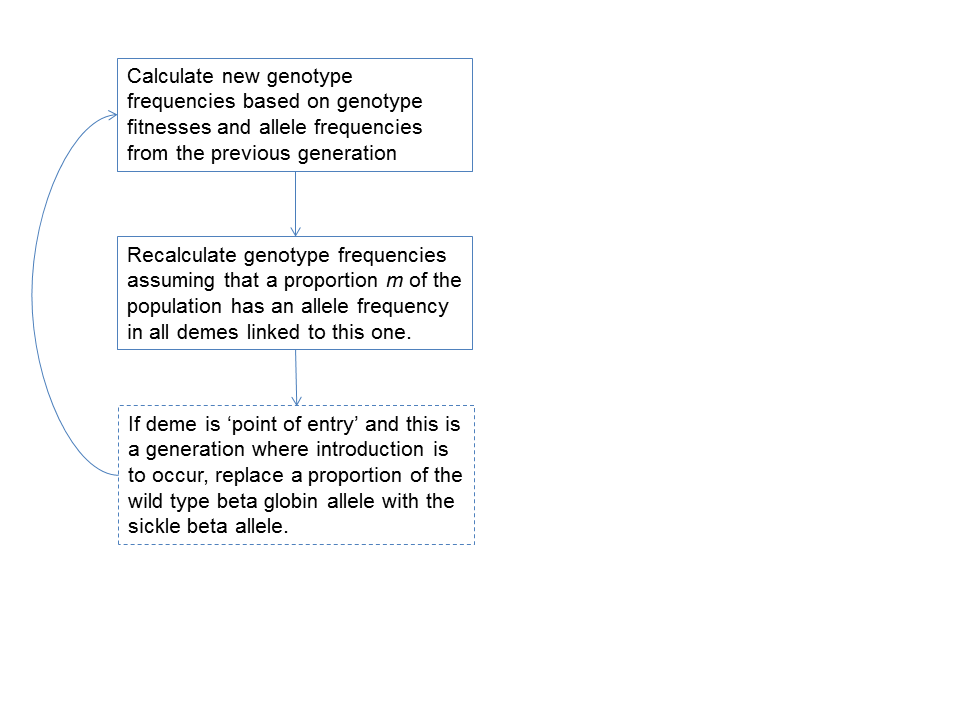


**Figure S2: Illustrating how the connectivity of the network was varied**

Panels (a)-(d) show examples of networks constructed with p=0; (b) p=0.1; (c) p=0.2 and (d) p=0.4.

The network diagrams in this figure were produced using Gephi (Bastian, M., Heymann, S., Jacomy,M., 2009).

**
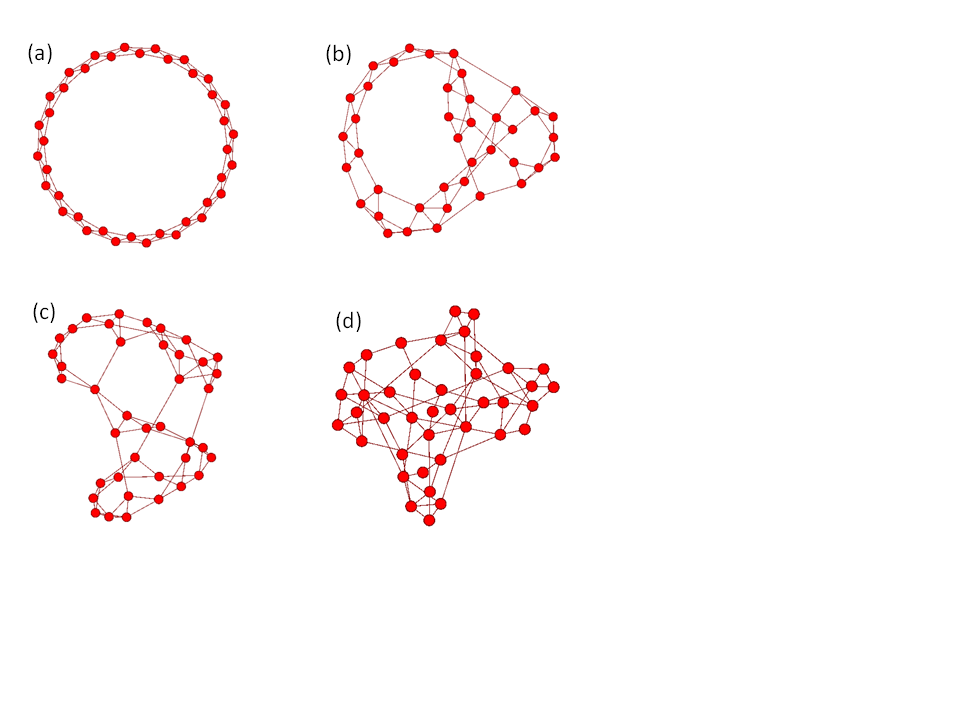
**

**Figure S3: Equilibrium behaviours for the mortality framework given in Table 1.** These are the results if malaria is assumed to exact an excess mortality of 0.005 years^-1^ on the population. In (a) β^S^ is introduced into a population containing low levels of the thalassaemias, and the ‘subSaharan’ outcome is obtained (alpha thalassaemia coexists with β^S^, but alpha thalassaemia’s frequency is kept relatively low). In (b) β^S^ is introduced into a population containing high levels of the thalassaemias (they each start at a frequency of 0.08) and β^S^ can never become established in the population.


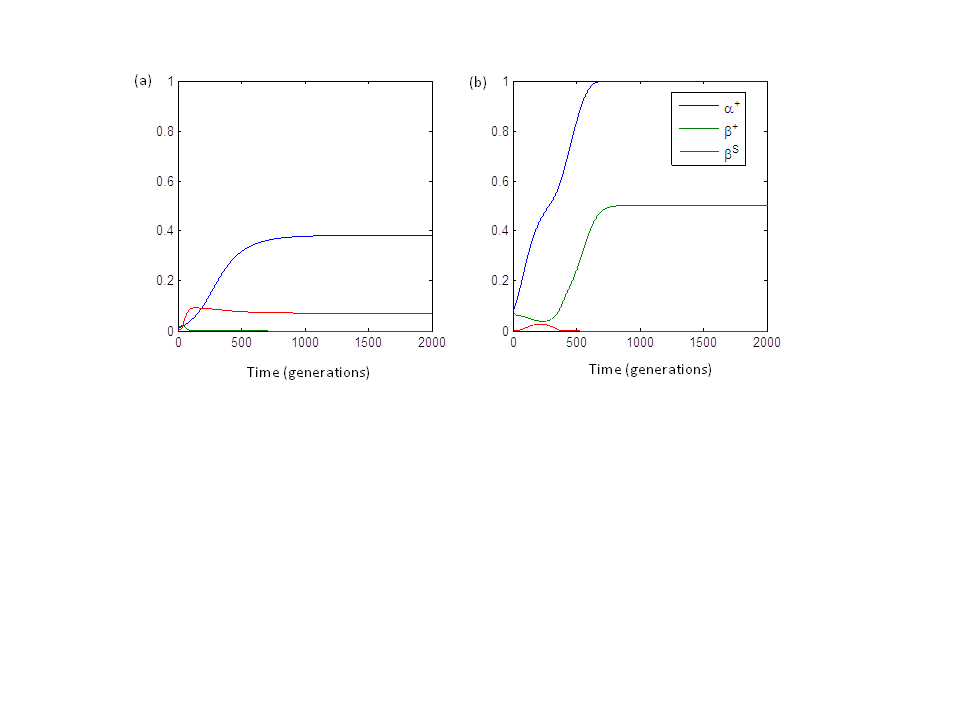


**Figure S4: The stratification of β^+^ alleles (defined by their blood disorder severity and protectiveness against malaria) by ability to resist invasion by β^s^.**  Panel (a) does not include positive epistasis between alpha and beta thalassaemia; panel (b) includes positive epistasis between alpha and beta thalassaemia. As discussed in the Methods and in the legend to table 1, negative epistasis between alpha thalassaemia and sickle cell trait was always included. The labels in the shaded areas refer to the starting frequencies of beta thalassaemia that would be required to resist invasion from a β^s^ allele introduced at a frequency of 0.0001, if the beta thalassaemic allele had the properties indicated by the x and y axes. For both panels, alpha thalassaemia had a starting frequency of 0.1 and the level of malaria selection was 0.005 years^-1^


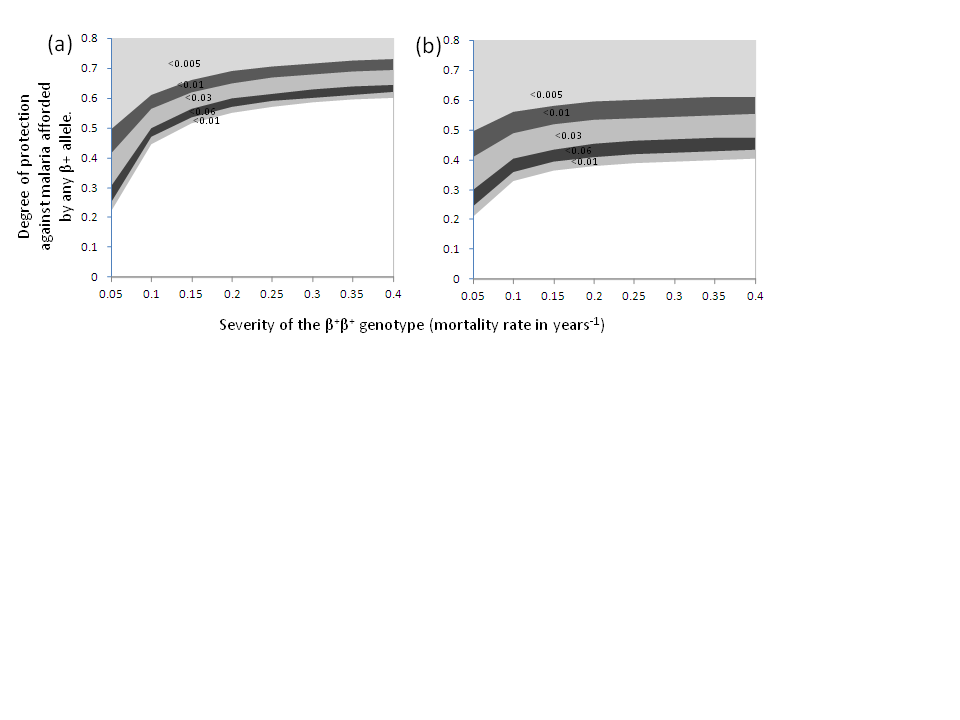


**Figure S5: the spread of β^S^ at different levels of gene flow.**  This figure illustrates how gene flow affects the spread of β^S^ at two different thalassaemia starting frequencies: 0.04 (panels a and c) and 0.08 (panels b and d). As in figure 2, malaria selection is applied to every deme at a level of 0.005 years^-1^, and after its first introduction, β^S^ is assumed to re-challenge the population in 30% of subsequent generations, chosen at random. Panels (a) and (b) illustrate the distribution of the proportion of demes where β^S^ < 0.005 after 100 generations, for 100 different repeats with each set of conditions. Panels (c) and (d) illustrate the distribution of mean frequencies of β^S^ across the whole metapopulation, for 100 different repeats with each set of conditions. Each boxplot shows the median (a dot within a circle); interquartile range (the box) and the range of values not considered outliers (the whiskers). Outliers are indicated by unfilled circles. We used the default settings in Matlab version 7.11.0, so outliers were any point that was greater than *q*_3_ + 1.5(*q*_3_ – *q*_1_) or smaller than *q*_1_ – 1.5(*q*_3_ – *q*_1_), where *q*_1_ and *q*_3_ are the 25th and 75th percentiles.

**
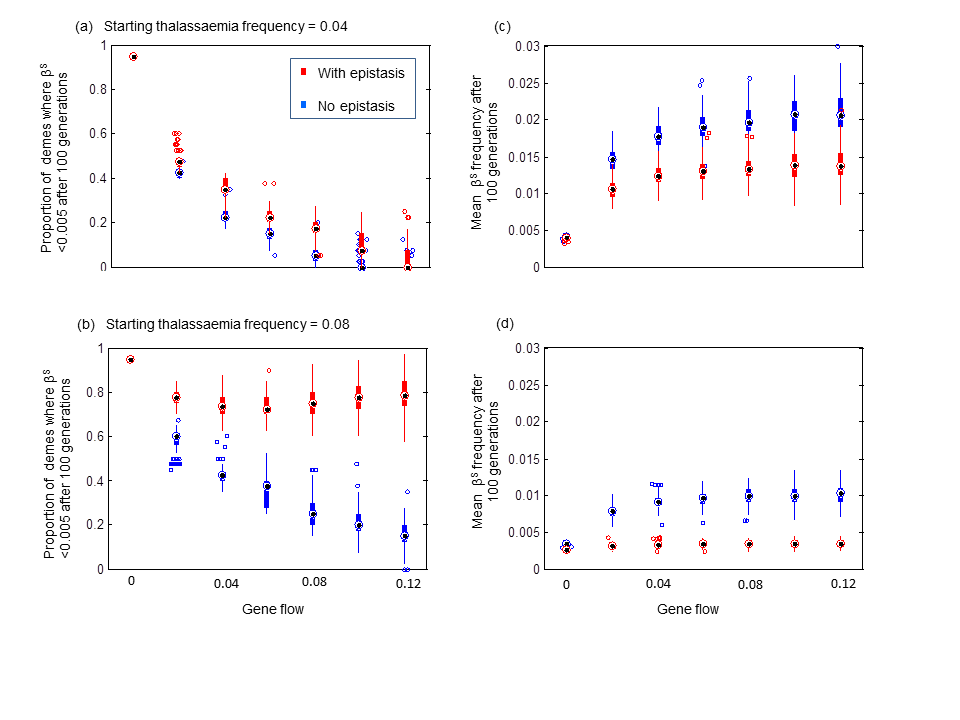
**

**Figure S6: allele frequencies over 100 generations**

The four panels indicate the changes in allele frequencies in deme 30 over 100 generations, for a scenario in which malaria exerted an excess mortality of 0.005 years^-1^; the starting frequency of the thalassaemias was 0.08; the level of mixing between demes was 0.1, and sickle cell was introduced at the beginning of the simulation, then in 30% of subsequent generations (chosen at random). Panels (a) and (b) illustrate alleles at the beta globin locus; panels (c) and (d) at the alpha globin locus.

**
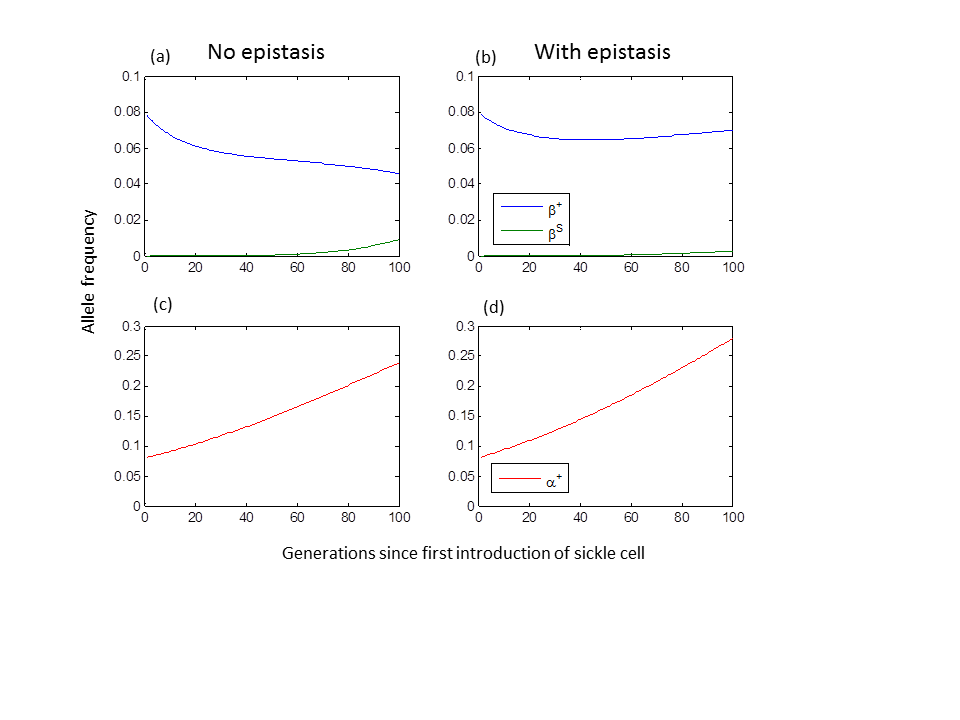
**

**Figure S7: the effect of network structure on the spread of sickle cell and formation of hotspots**

At each value of p between 0.1 and 0.8 we tested 30 different network structures. We also tested the network when p=0 (only one such network is possible). The results are summarised in the following panels. Scenarios where positive epistasis between alpha and beta thalassaemia has been included are shown in red; scenarios without such epistasis are shown in blue. The thalassaemias always had a starting frequency of 0.08, and simulations ran for 100 generations with a malaria selection level of 0.005 years^-1^. Each boxplot shows the median (a dot within a circle); interquartile range (the box) and the range of values not considered outliers (the whiskers). Outliers are indicated by unfilled circles. We used the default settings in Matlab version 7.11.0, so outliers were any point that was greater than *q*_3_ + 1.5(*q*_3_ – *q*_1_) or smaller than *q*_1_ – 1.5(*q*_3_ – *q*_1_), where *q*_1_ and *q*_3_ are the 25th and 75th percentiles.

**Figure S8: the impact of epistasis on the formation of hotspots under different conditions**

This figure illustrates the mean number of hotspots observed over 100 simulations (y axis), with different intensities of sickle cell challenge (x axis). The error bars indicate ± 2 standard errors of the mean. The extra mortality exacted by malaria was 0.005 years^-1^.

**
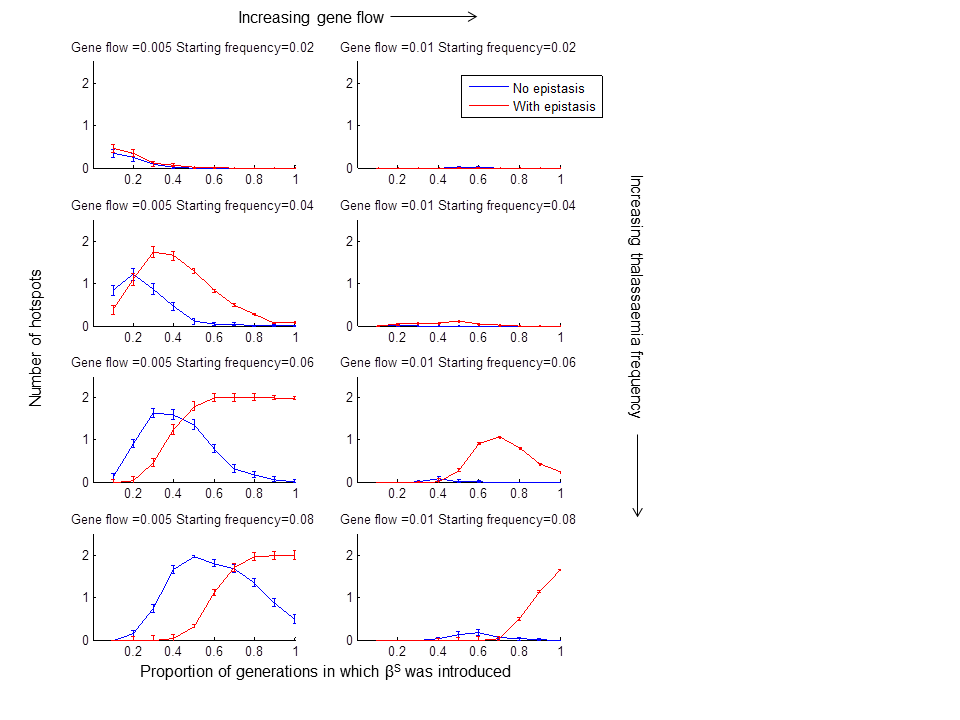
**

**Figure S9: A two-community network and the formation of hotspots**

Panel (a) illustrates the simplified network that we developed to explore how β^S^ spreads between two communities linked by varying degrees of gene flow. Panel (b) indicates the mean frequency of β^S^ in community two after 100 generations, and panel (c) indicates the ratio of β^S^ frequencies in community one to β^S^ frequencies in community two after 100 generations. Within-community gene flow was 0.15 and malaria selection was set at 0.005 years^-1^. The initial frequency of alpha thalassaemia in all demes was 0.09; the initial frequency of beta thalassaemia was 0.11 and that of β^S^ was 0, except in the deme indicated by the arrow in which the starting frequency of beta thalassaemia was 0 and the starting frequency of β^S^ was 0.03. This model was entirely deterministic: we seeded a single deme with β^S^ and did not re-challenge the population.


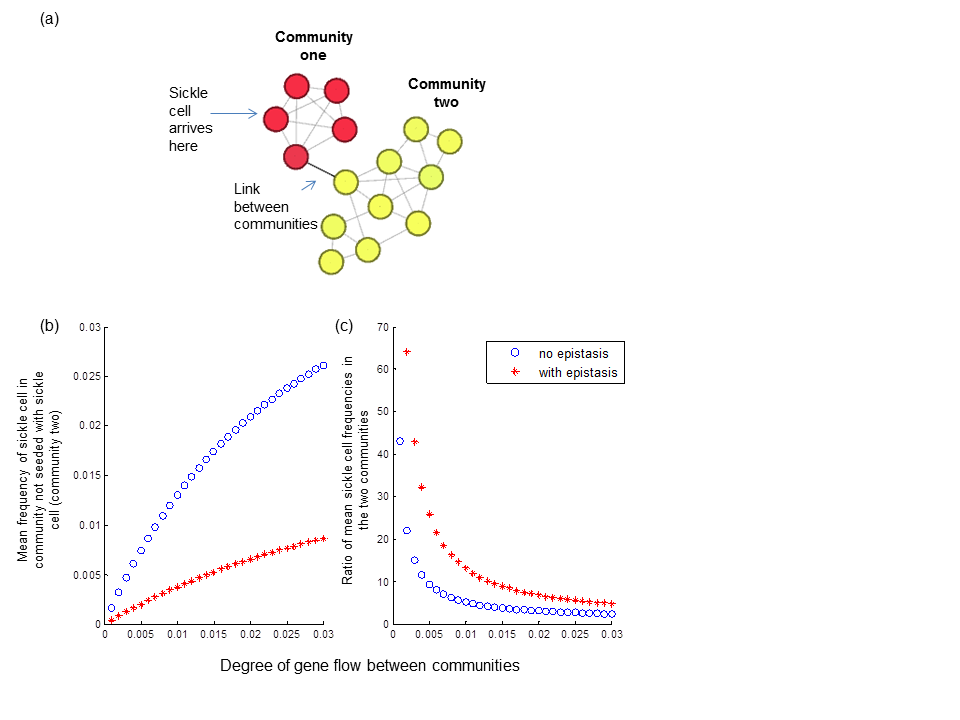

Supplement: Supplementary data 1 [file mmc1.docx]
